# Medical practitioners' reactions towards family medicine as a speciality in South Africa

**DOI:** 10.4102/phcfm.v1i1.11

**Published:** 2009-04-28

**Authors:** Cyril Naidoo, Tonya Esterhuizen, Prem Gathiram

**Affiliations:** 1 Department of Family Medicine, University of Kwa-Zulu Natal, South Africa; 2 Department of Biostatistics, University of Kwa-Zulu Natal, South Africa

**Keywords:** family medicine, family physicians, medical practitioners, reactions, speciality

## Abstract

**Background:**

Family physicians are trained to treat a wide range of diseases, treatment being centred on the patient, family and community irrespective of age, gender, or ethnic or racial background. To deal with inequalities in health care, the South African government introduced the concept of a district health system in 1997. It was only in August 2007, however, that family medicine was legislated as a speciality. This study was undertaken prior to the enactment of this legislation.

**Method:**

A descriptive quantitative study using a self-administered questionnaire was undertaken. A convenience sampling technique was used (N = 60) to assess the reactions of medical practitioners towards the impending legislation.

**Results:**

Overall, 60% of the sample was in favour of the legislation. There were no significant differences between those working in the private and public sectors or between generalists and specialists. With regard to those not in favour of the legislation compared to those in favour of the legislation, a significantly increased number answered the following statements in the affirmative: (i) ‘I already carry out the functions of a family physician’ (p = 0.001), (ii) ‘They [specialist family physicians] will not be as qualified as specialists in other categories’ (p = 0.005), (iii) ‘It will have a negative impact on general practice’ (p < 0.001), (iv) ‘It will increase competitiveness’ (p = 0.021), (v) ‘It will not have any effect on patient care’ (p = 0.010) and (vi) ‘There is no need for such a speciality’ (p = 0.001).

**Conclusion:**

We concluded that the majority were in favour of the legislation being implemented.

## INTRODUCTION

In many countries, generalists and medical students are encouraged to specialise in family medicine or a similar primary care speciality to address the perceived decline in general practice.^1–7^ In the USA, family medicine speciality has, in fact, been in existence since 1969^8^. Family physicians are the only medical specialists who are trained to treat patients holistically and who have the necessary generalist skills to treat most ailments. They are expected to provide personal, comprehensive, frontline and continuing health care for people of all socio-economic strata, all ages and both sexes, irrespective of ethnicity.^7, 9^ To improve and support the health of all the nations of the world, the World Health Organisation has called for the development of a primary health care system.^10^ This would also help to deal with global inequalities in health and would provide an effective, sustainable health system and access to quality services for acute and chronic diseases.

In order to move towards a primary care approach to health in South Africa, the government introduced the district health system in 1997.^11^ It was envisaged that family physicians would be the main roleplayers driving the delivery of health to all people at this level.^11^ Even though this legislation allowed the opening of a register for family physicians by the Health Professions Council of South Africa in 1993, however, it was only in August 2007 that the national Department of Health officially recognised family medicine as a speciality.^11, 12^

In most countries where the category of family medicine is recognised as a speciality, health care is nationalised and there is thus no competition for patients with other specialities. South Africa, however, has a two-tiered system (public and private); there is thus always the potential for competition for patients within the private sector. It therefore becomes difficult to predict the attitudes and perceptions of medical doctors, especially in the private sector, towards such a speciality.

With this in mind, we investigated, just prior to the promulgation of the Act, the views of general practitioners and specialist physicians in internal medicine about the new legislation.

## METHOD

A descriptive quantitative study was conducted using a self-administered questionnaire to assess the perceptions and attitudes of medical practitioners towards the imminent promulgation of the category of family medicine as a speciality by the national Department of Health. Approval to conduct this study was obtained from the Ethics Committee of the University of KwaZulu-Natal. The questionnaire was first piloted among general practitioners and specialists at a hospital in order to eliminate any ambiguity. Participants in the pilot study were not included in the study.

**Study population:** The study population consisted of general practitioners and specialist physicians from both private and public health sectors within the eThekwini metropolitan area, Durban, South Africa.

**Sample:** A convenience sample of 60 was used. The participants were enrolled from one large public hospital and two private doctor groupings.

**Inclusion criteria:** Inclusion criteria consisted of all general practitioners and general specialists in internal medicine practising in the private and public health sectors.

**Exclusion criteria:** Sub-specialists in the categories of cardiology, of gastroenterology, of neurology, of neurosurgery, of anaesthetics and of cardio-thoracic surgery, family physicians with MMed(Fam Med) and MFCP qualifications, and current students registered for the latter were excluded.

Prior to the administration of the questionnaire at the selected hospital and at the doctors’ guild meetings, all general practitioners and general specialists in internal medicine were fully informed of all the aspects of the study and that they had to complete a self-administered questionnaire in which, in order to maintain anonymity and confidentiality, they were not required to indicate their name or any contact details. In addition, they were given a copy of the information document, which was a summary of the research project. Those willing to participate were required to sign an informed consent form.

**Statistical analysis:** SPSS version 15.0 was used to capture and analyse the data. Bivariate associations of variables were examined using Pearson's chi-square tests. A p value of < 0.05 was considered statistically significant.

## RESULTS

Of the 60 participants, 47 were male and 13 were female. Overall, 36 (60%) approved of the new legislation, while 15 (25%) disagreed and 9 (15%) were undecided (Table 1). There was no statistical difference between male and female respondents in terms of their approval of the legislation (p = 0.653). As shown in Table 1, 57.4% of males and 69.2% of females were in favour of the legislation. Experience in practice was not associated with the approval of the legislation either (p = 0.449). Fifty percent of the participants had been in practice for more than 20 years and the majority of them (60%) was in favour of the legislation. Interestingly, 60% (N = 18) of those who were in practice for less than 20 years also came out in support of the legislation.

**TABLE 1:** Number and percentage of medical practitioners in favour of or against the legislation by gender and years in practice

		APPROVE OF LEGISLATION						P VALUE

		YES		NO		UNDECIDED		
		
		n	%	n	%	n	%
**Gender**	Male (n = 47)	27	57.4%	13	27.7%	7	14.9%	0.653
	Female (n = 13)	9	69.2%	2	15.4%	2	15.4%	
**Years in practice**	< 20 years	18	60%	6	20%	6	20%	0.449
	> 20 years	18	60%	9	30%	3	10%	

**FIGURE 1:**
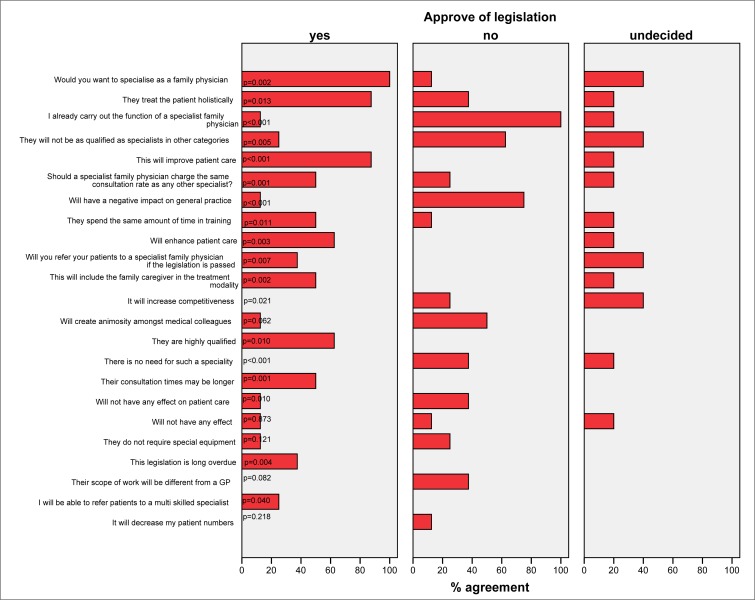
Bar chart showing percentages of who approved and disapproved of the legislation by agreement with the listed statements or questions

Table 2 and Figure 1 compare the responses to a number of listed questions and statements between those who agree (‘yes’), disagree (‘no’) or were undecided towards the legislation. The percentages shown are column percentages of those who agreed with the listed statement or question. There was a highly significant difference in responses to almost all the statements and questions between those who approved and those who disapproved of the legislation. The question on which respondents agreed most was: ‘Would you like to specialise as a family physician?’ (A total of 57.4% answered ‘yes’ to this question.) Agreement was highest in those who approved of the new legislation compared to those against (p = 0.002). A higher proportion of respondents who agreed with the legislation were of the view that the legislation was long overdue (p = 0.004).

**TABLE 2:** Differences in responses (%) to various statements between those in favour (‘yes’) compared to those not in favour (‘no’) of the legislation

	APPROVE OF LEGISLATION			P VALUE

	YES	NO	UNDECIDED	
Would you want to specialise as a family physician?	77.40%	6.70%	11.10%	0.002
Should a specialist family physician charge the same consultation rate as any other specialist?	75.00%	0.00%	22.20%	0.001
This will enhance patient care.	70.60%	0.00%	0.00%	0.003
This will improve patient care.	68.60%	33.30%	11.10%	< 0.001
They treat the patient holistically.	61.10%	20.00%	11.10%	0.013
Will you refer your patients to a specialist family physician if the legislation is passed?	60.00%	53.30%	22.20%	0.007
This will include the family caregiver in the treatment modality.	50.00%	33.30%	22.20%	0.002
They spend the same amount of time in training.	44.40%	33.30%	12.50%	0.011
Their consultation times may be longer.	41.70%	50.00%	0.00%	0.001
They are highly qualified.	41.70%	33.30%	25.00%	0.010
This legislation is long overdue.	36.10%	20.00%	16.70%	0.004
I will be able to refer patients to a multiskilled specialist.	30.60%	22.20%	33.30%	0.040
This will create animosity among medical colleagues.	18.20%	80.00%	25.00%	0.062
They will not be as qualified as specialists in other categories.	11.10%	6.70%	50.00%	0.005
This will not have any effect on patient care.	9.10%	40.00%	22.20%	0.010
I already carry out the function of a specialist family physician.	8.30%	13.30%	33.30%	< 0.001
Their scope of work will be different from that of a GP.	8.30%	6.70%	0.00%	0.082
This will have a negative impact on general practice.	4.50%	80.00%	22.20%	< 0.001
It will increase competitiveness.	2.80%	6.70%	0.00%	0.021
They do not require special equipment.	2.80%	0.00%	0.00%	0.121
It will decrease my patient numbers.	0.00%	6.70%	22.20%	0.218
There is no need for such a speciality.	0.00%	0.00%	22.20%	< 0.001

Of those who were opposed to the legislation compared to those in favour, a significantly greater proportion were of the view that (i) they were already carrying out the functions of a family physician (p = 0.001), (ii) they [specialist family physicians] would not be as qualified as specialists in other categories (p = 0.005), (iii) the legislation would have a negative impact on general practice (p < 0.001), (iv) the legislation would increase competitiveness (p = 0.021), (v) the legislation would not have any effect on patient care (p = 0.010) and (vi) there would be no need for such a speciality (p < 0.001). A small but insignificant number felt that their number of patients would be reduced should the legislation be enacted.

There was furthermore no statistically significant difference in approval among the different health sectors because the majority of participants from the private (60%), the public (69%) and the private/public partnership (67%) sectors were in favour of the legislation.

## DISCUSSION

In most countries where the health system has been nationalised, the introduction of family medicine as a speciality did not pose a problem at the time of doing so. This is the first study conducted to test the attitudes and perceptions of medical practitioners on the promulgation of family medicine as a speciality in South Africa, a country where the health system is still not nationalised, even though the state does provide health care facilities for the entire population.

This study shows that most of the respondents (both generalists and specialists) favoured the promulgation of the legislation making family medicine a speciality in South Africa. A significant number of generalist practitioners was in favour of the legislation and indicated a desire to specialise in family medicine. Those who were not in favour of family medicine being regarded as a speciality were of the attitude (or perhaps had the perception) that it would have a negative impact on their practice because it would introduce competitiveness and – even worse – that there was no need for such a speciality (Table 2). These respondents were furthermore under the impression that they were already carrying out the functions of a family physician and that such specialists would not be as qualified as specialists in other categories. The latter two responses perhaps illustrate the fact that the participants were not aware of the scope, extent and nature of the training of family medicine specialists.

In sub-Saharan Africa, which has some of the world's poorest communities and very high death rates from preventable infectious diseases compared to the developed world, the primary health care system has not developed as one would have anticipated.^13^ In most countries outside Africa, family medicine specialists play an important role in the treatment of the general populace.^14–22^ In most public hospitals in sub- Saharan Africa, however, there are fewer family physicians, in other words, there is a low doctor-to-patient ratio.^13^ This, together with a large number of vacant medical officers’ posts, contributes to increased patient congestion and the perceived poor health care.^23^ To compound the problem, family physicians are a scarce skills force in many poorer countries in Africa, where the first line of contact with patients is usually nurses or physician assistants or even traditional healers.^24^ Family physicians, where present, serve as generalist physicians or even emergency surgeons rather than concentrating on family and preventative medicine.^24^

As far as we are aware, besides South Africa, Nigeria^25^ and Kenya^26^ are the only two other countries in Africa that offer family medicine speciality (in Nigeria, this is known as ‘general practice’). Although family medicine is regarded as a speciality in Egypt, the training of practitioners is undertaken outside Africa.^27^ In Uganda and perhaps other sub-Saharan countries (excluding South Africa), the ministries of health have not clarified the exact role that family physicians should play in the health care system.^28^ This uncertainty has resulted in fewer medical graduates wanting to specialise in family medicine.^28^ Fortunately, in South Africa, each medical school has a department of family medicine, which offers postgraduate and Master's degrees in family medicine. Family medicine has furthermore been part of the undergraduate curriculum for many years.^11, 29^ The integration of family medicine into the mainstream primary health care system in many of the hospitals in the country may therefore not be difficult to implement following said legislation. Registrar posts in family medicine have furthermore already been allocated to hospitals that have well-developed family medicine departments. It is envisaged that the core curriculum and procedural skills required for the family medicine speciality will be similar to those of the USA and Canada^30, 31^ and other parts of the world. In other words, family medicine registrars will be expected to rotate through internal medicine, anaesthetics, paediatrics, obstetrics and gynaecology, and mental health disciplines and to exit with competencies in a wide range of core clinical and procedural skills.

It is perhaps still too early to predict the role that family medicine specialists will play within the primary health care system in South Africa and whether there will be sufficient demand from medical graduates to take up this speciality in the years to come, as is experienced in many countries.^32–35^ Academic departments are nevertheless quite optimistic that family medicine specialists will play an important role in both urban and rural primary health care because, in hospitals where family medicine departments already exist, family physicians are not only contributing towards the upliftment of health care in general but also training other health care professionals, such as nurses and junior medical staff.

Although this study shows that most participants were in agreement that family medicine be recognised as a speciality, they felt that some of the problems that may arise, as has happened elsewhere, may be related to remuneration (if family medicine specialists are paid a lower salary than specialists in other categories),^1, 36^ to increased hours of work (which may result in burn-out),^37–39^ to a lack of autonomy or to powerlessness in their workplace, and to management's inability to recognise the important role that family physicians play in making appropriate decisions with respect to complex medical conditions.^39^

### Limitations of the study

This study has certain limitations, the main one being that the required number of participants (specialists and generalists alike) from the private and public sectors could not be recruited into the study because, in the midst of the study, the legislation making family medicine a speciality was promulgated and gazetted. This resulted in the study being terminated prematurely, hence the smaller number of specialist participants and the discrepancies in the number of participants from the private and public sectors possibly skewing the outcomes. Because a convenience sampling method was used, it is difficult to generalise the data as the views of general practitioners and specialist physicians from the country as a whole. We do recommend, however, that a broader study be undertaken throughout the country to re-evaluate the perceptions and attitudes of generalists and specialists in internal medicine and other disciplines both from the public and the private sectors towards family medicine as a speciality.

### Conclusion

We conclude from the findings of this study that the majority of the participants was in favour of family medicine being recognised as a speciality
